# Comparisons of Characteristics Between Psychological Support Hotline Callers With and Without COVID-19 Related Psychological Problems in China

**DOI:** 10.3389/fpsyt.2021.648974

**Published:** 2021-05-12

**Authors:** Liting Zhao, Ziyang Li, Yongsheng Tong, Mengjie Wu, Cuiling Wang, Yuehua Wang, Nancy H. Liu

**Affiliations:** ^1^Beijing Suicide Research and Prevention Center, Beijing Huilongguan Hospital, Beijing, China; ^2^World Health Organization Collaborating Center for Research and Training in Suicide Prevention, Beijing, China; ^3^Peking University Huilongguan Clinical Medical School, Beijing, China; ^4^Department of Psychology, University of California, Berkeley, Berkely, CA, United States

**Keywords:** COVID-19, psychological problem, hotline, psychological intervention, suicide

## Abstract

**Background:** To compare the characteristics between hotline callers with and without the Coronavirus Disease 2019 (COVID-19) related psychological problems.

**Methods:** From January 25 to March 31, 2020, 581 callers with COVID-19 related psychological problems (COVID-19 callers) and 695 callers without COVID-19 related psychological problems (non-COVID-19 callers) to the Beijing Psychological Support Hotline were recruited. The demographic characteristics, primary concerns, suicidal ideation, depression and other psychological problems were compared between the two groups of callers.

**Results:** Both groups of the callers were predominantly female and highly educated. The primary concerns reported by the COVID-19 callers were depression (38.4%) and family relationship problems (26.0%). As compared to the non-COVID-19 callers, COVID-19 callers reported more financial (7.4%) and work related problems (4.1%), but revealed lower prevalence of suicidal ideation (47.9% v 71.3%), lower degrees of psychological distress (74.3 v 79.1), intensity of suicidal ideation (0 v 50), severity of depression (57.9 v 65.1), and higher degree of hopefulness (41.1 v 33.6) (all *p* values < 0.01). Additionally, a lower proportion of COVID-19 callers met the criteria of depressed mood (51.6% v 61.4%) and other 4 symptoms than the non-COVID-19 callers (*p* values < 0.01).

**Conclusions:** Based on the content of the primary concerns and the relatively low level of depression of the COVID-19 callers, the psychological intervention for them during the pandemic should focus on “psychological supports.” Coping strategies for daily life stressors and promotion of scientific knowledge about the pandemic should also be included in the hotline-related interventions.

## Introduction

The outbreak of the Coronavirus Disease 2019 (COVID-19) has had a substantial impact on the mental health of the general population (1–4). During the pandemic, confirmed cases, people in quarantine, front-line healthcare workers and the general public have experienced varying degrees of anxiety, distress, and fear (2). To mitigate the psychological disturbance and possible psychological damage to the public, various forms of professional psychological crisis intervention services have been delivered in China (5). Our psychological support hotline, an online mental health service, provides real-time interactive psychological support, guidance, and crisis intervention remotely to different groups of people (6, 7). During the pandemic, the Beijing Psychological Support Hotline (BPSH) provides 24/7 COVID-19 related psychological counseling services to Mandarin-speaking Chinese globally.

The psychological support hotline is considered to play a key role in responding to public emergencies (8, 9). Most of the previous studies about hotline callers have focused on the general characteristics of callers and effectiveness of interventions for suicide (10–13). During the 2003 outbreak of the Severe Acute Respiratory Syndrome (SARS), a preliminary study on the characteristics of the callers to the epidemic psychological support hotline in China concluded that callers with epidemic related problems were predominantly female, middle-aged and young adults, with main concerns about mood and SARS-related questions (14, 15).

Although a large number of studies have reported the impact of COVID-19 on the mental health of the public (3, 4, 16, 17), many individuals had mental health problems prior to the pandemic or their concerns were unrelated with the COVID-19. Thus, it is improper to indiscriminately deliver psychological crisis intervention services to hotline callers, disregarding whether their main concerns were COVID-19 related or not. In order to understand the impact of the pandemic on public mental health, we compare characteristics of psychological disturbances between the callers whose concerns were and were not COVID-19-related. These findings will be useful for the further development of more specific hotline-based psychological crisis intervention model during public health emergency.

During the COVID-19 pandemic, the BPSH received a large number of calls with psychological problems related to the disease. The present study aims to analyze the probable differences between the hotline callers who reported psychological problems associated with COVID-19 (COVID-19 calls) and those with psychological problems unrelated with the pandemic (referred to as “non-COVID-19 calls”). Based on BPSH data, we focus on the probable differences in the demographic characteristics, primary concerns, suicidal ideation, depression and other psychological problems between the two groups of callers during the most severe period of COVID-19 in China.

## Materials and Methods

### Sampling

Shortly after the announcement of the human to human contagion of the COVID-19 on January 20th, 2020, the BPSH labeled each call as COVID-19 or a non-COVID-19 call. If caller complained that his/her psychological disturbances were related to the COVID-19, or mentioned COVID-19 more than once during the hotline conversation, the call was labeled as a COVID-19 call. Whereas, if the caller did not mention the epidemic at all during the entire call, it was determined as a non-COVID-19 call.

All calls to the BPSH during January 25th to 31st March 2020—the most serious stage of the epidemic in China—were considered for the present study. Exclusion criteria were: (1) “null” calls, (i.e., silence only or hoax callers; (2) the caller's main purpose was not seeking for psychological support, (3) repeat calls (i.e., multiple calls from the same person, reported by callers or indicated by phone number). For repeat calls, only one call was selected for analysis. Generally, the call with the fewest missing interested data was selected; in the case that the number of variables with missed data was equal for repeated calls, the first call was selected. Among the calls which met the above criteria, all COVID-19 calls were included. Given many more non-COVID-19 calls were expected during the study period, we randomly selected (using SPSS 18.0) 20% of the eligible calls in the final data analysis.

### Measures

At the BPSH, operators are required to follow a specific work-flow and ask callers for demographic information, including gender, age, education in years, marital status, and work status. In addition, operators ask callers about their suicidal ideation and the intensity of the ideation (0–100 points), their degree of psychological distress (on a scale of 0–100, with 0 meaning no psychological distress and 100 meaning the most severe psychological distress), as well as their hopefulness score (on a scale of 0–100, with 0 meaning completely hopeless and 100 meaning completely hopeful). Similarly, a score of 0 is regarded as without suicidal ideation and 100 means that one definitely wants to take one's life. The above assessment is performed twice per call, i.e., at the beginning and at the end of the index call.

The primary concerns reported by callers are categorized into nine groups: (1) family relationship problems, referring to conflicts with family members; (2) non-family relationship problems, referring to interpersonal conflicts peoples other than family members, including romantic relationship breakup; (3) financial problems, referring to debts, failed investments, etc.; (4) work-related problems; (5) school or study-related problems; (6) other negative life events; (7) psychiatric problems, defined as a history of any mental disorder other than depression; (8) depression, referring to severe depression as detected by the structured Chinese Depression Screening Scale (18); and (9) other problems, i.e., areas that could not be specifically categorized into the above eight problems. At the end of the call, the operator selects no more than the top three categories from which to record the primary concerns that best reflect the caller's psychological situation.

#### Suicidal Ideation and Plan

Suicidal ideation and plan are assessed by the operator asking the caller, “In the last 2 weeks, have you repeatedly thought about death, felt that death is better than living, or thought about hurting yourself?” If the caller responds “yes,” the caller will then be asked if there is an actual suicide plan. Based on the caller's response, the operator classifies the caller as one of the following three statuses: no suicidal ideation, suicidal ideation without a specific plan, or suicidal ideation with a specific plan.

#### Depression

The presence of 9 depressive symptoms of the Diagnostic and Statistical Manual of Mental Disorders, and the duration of the symptoms (if present) are assessed by the operator using the structured Chinese Depression Screening Scale (18). The score for depressive symptoms is the product of severity and days, summed for the 9 depressive symptoms. Then the score is converted into 0–100. The eight depressive symptoms other than suicidal ideation (classified as either present or absent) are classified into three levels: symptomatic (i.e., symptoms were present for at least 14 days); subthreshold symptoms (i.e., symptoms were present but for <14 days); or asymptomatic (i.e., symptoms were not present).

#### Other Social and Psychological Variables

Other psychological problems were defined as the following: (1) history of prior suicide attempt; (2) substance misuse; (3) chronic life events, i.e., long-term and current adverse psychological effects of past or current life events, such as those with ongoing family conflicts or work stress; (4) acute life events; (5) history of physical/sexual abuse; (6) fear of being attacked in the past month; (7) severe physical illness, i.e., presence of physical illness or disabilities that have a serious impact on their lives; and (8) history of suicidal acts of family members or friends.

These psychological problems were assessed by the operator asking the caller one by one, following preset instructions. For example, presence of acute life events is assessed by asking the caller, “In the last week, have any life events happened that seriously affected you psychologically?” If the caller answers “yes,” he/she would be further asked to evaluate the severity of the impact (on a scale of 1–5, with no effect counted as one and a maximum effect counted as five). A score of 3 (moderate effect) or higher was considered as experiencing an acute life event.

### Statistical Analysis

In this study, age and education in years were converted into tertiles; marital status was classified as unmarried, married, and others; and employment was classified as student, employed, unemployed, and other. The changes in the caller's psychological distress, hopefulness, and intensity of suicidal ideation before and after the call were the difference between the beginning and the ending of the call. Chi-square tests, independent samples *t*-tests, and Mann-Whitney U tests were used to compare the differences between COVID-19 callers and non-COVID-19 callers.

## Results

**Figure 1:**
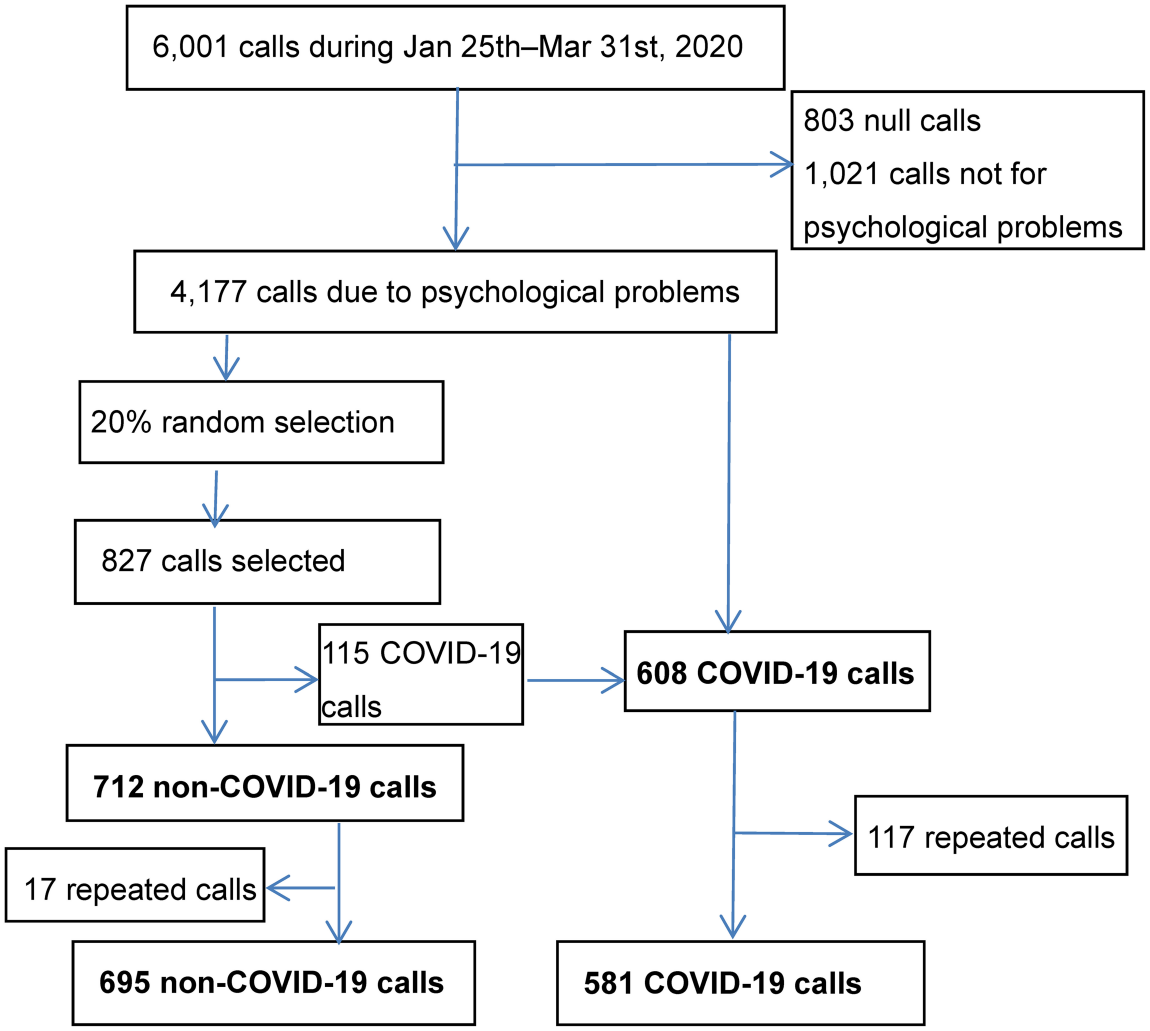
Flow chart for recruitment of the call.

The process of sampling is shown in the Figure 1. Briefly, the BPSH received 6,001 calls from January 25th to 31st March 2020. Eighteen percent of calls were from Beijing, 3.6% of calls from Hubei Province, calls from other provinces varied between 0.1–7.0%, and the other 0.3% of calls from overseas including Taiwan, Hongkong, and Macao. A total of 803 calls identified as null (e.g., silence only, hoax calls) and 1,021 calls not seeking psychological support were excluded. The final sample was 4,177 calls seeking psychological support. Among these, 827 calls were randomly selected. One hundred and fifteen of the 827 calls were COVID-19 calls, thus remained 712 calls were non-COVID-19 callers. Repeat calls were excluded, resulting in 695 non-repeat non-COVID-19 calls. Meanwhile drawing from the original full sample, 581 non-repeat COVID-19 calls were also identified and included.

The 1,276 recruited calls averaged 44.2 min in length of the call, with 45.9 min for COVID-19 calls and 42.9 min for non-COVID-19 calls. As seen in Table 1, 66.2% of the callers were female, and the gender difference between the COVID-19 callers and non-COVID-19 callers was not statistically significant. There were however, statistically significant differences in demographic variables such as age, education years, marital status, and employment status between the two groups. More than twice as many of COVID-19 callers were over 30 years old as that in the non-COVID-19 callers. COVID-19 callers were more highly educated, more likely to be married, and were employed than non-COVID-19 callers.

**Table 1:** Comparison of characteristics of COVID-19 callers and non-COVID-19 callers [*n* (%)].

**Characteristics**	**All callers**	**COVID-19 callers**	**Non-COVID-19 callers**	*****χ^2^*****	***p***
	**(*n* = 1,276)**	**(*n* = 581)**	**(*n* = 695)**		
Gender				0.02	0.896
Female	844 (66.2)	386 (66.4)	458 (66.1)		
Male	430 (33.8)	195 (33.6)	235 (33.9)		
Age				110.80	<0.001
<20 years	436 (35.7)	132 (23.3)	304 (46.3)		
20–29 years	480 (39.2)	221 (39.0)	259 (39.4)		
30+ years	307 (25.1)	213 (37.6)	94 (14.3)		
Education years				45.39	<0.001
0–9	371 (30.9)	125 (22.6)	246 (37.9)		
10–12	250 (20.8)	105 (19.0)	145 (22.3)		
≥13	580 (48.3)	322 (58.3)	258 (39.8)		
Marital status				73.99	<0.001
Unmarried	963 (78.1)	380 (67.1)	583 (87.4)		
Married	219 (17.8)	153 (27.0)	66 (9.9)		
Other	51 (4.1)	33 (5.8)	18 (2.7)		
Employment status				90.43	<0.001
Student	526 (43.0)	174 (30.6)	352 (53.8)		
Employed	459 (37.6)	278 (48.9)	181 (27.7)		
Unemployed	200 (16.4)	87 (15.3)	113 (17.3)		
Other	37 (3.0)	29 (5.1)	8 (1.2)		

*Each variable contains missing values, so the sum of the callers of each variable is less than the total number of callers*.

As seen in Table 2, the differences between the COVID-19 and non-COVID-19 groups were statistically significant for several groups of the primary concerns encountered by the callers. For COVID-19 callers, the top three primary concerns were depression, family relationship problems, and other psychiatric problems, while for non-COVID-19 callers, the top three major problems were family relationship problems, non-family relationship problems, and depression. The proportion of COVID-19 callers with family and non-family relationship problems was lower than that of non-COVID-19 callers, while the prevalence of depression, encountering financial and work-related problems among COVID-19 callers were higher than that of non-COVID-19 callers. While we subdivided the mentioned groups of primary concerns into specific stressors, results indicated that, COVID-19 callers were less likely to report conflicts with parents (16.0 vs. 24.7%, χ^2^ = 14.70, *P* < 0.001) and romantic relationship breakup (7.4 vs. 17.0%, χ^2^ = 26.33, *P* < 0.001) than non-COVID-19 callers, however, COVID-19 callers were more likely to experience high work-related competition (2.6 vs. 0.7%, χ^2^ = 7.11, *P* = 0.008) and income decrease (1.5 vs. 0.4%, χ^2^ = 4.24, *P* = 0.039) than non-COVID-19 callers.

**Table 2:** Comparison of the primary concerns reported by COVID-19 callers and non-COVID-19 callers [*n* (%)].

**Primary concerns**	**All callers**	**COVID-19 callers**	**Non-COVID-19 callers**	*****χ^2^*****	***p***
	**(*n* = 1,276)**	**(*n* = 581)**	**(*n* = 695)**		
Family relationship problems	370 (29.0)	151 (26.0)	219 (31.5)	4.69	0.030
Non-family relationship problems	255 (20.0)	74 (12.7)	181 (26.0)	35.04	<0.001
Financial problems	75 (5.9)	43 (7.4)	32 (4.6)	4.47	0.034
Work-related problems	66 (5.2)	41 (7.1)	25 (3.6)	7.72	0.005
Study-related problems	82 (6.4)	34 (5.9)	48 (6.9)	0.59	0.444
Other negative events	54 (4.2)	28 (4.8)	26 (3.7)	0.91	0.341
Depression (assessed)	386 (30.3)	223 (38.4)	163 (23.5)	33.43	<0.001
Other psychiatric problems	242 (19.0)	111 (19.1)	131 (18.8)	0.01	0.907
Other problems	13 (1.0)	5 (0.9)	8 (1.2)	0.27	0.607

Table 3 shows that the prevalence of suicidal ideation in COVID-19 callers in the 2 weeks prior to the index call was lower than those in the non-COVID-19 callers and reached statistical significance. As regards the proportion of callers with other social and psychological characteristics, the COVID-19 callers were less likely to report chronic life events, history of suicidal behavior, and fear of being assaulted than the non-COVID-19 callers. With respect to scores assessed at the beginning of the index call, COVID-19 callers reported lower scores of psychological distress, intensity of suicidal ideation, and severity of depression, but higher score of hopefulness than non-COVID-19 callers.

**Table 3:** Comparison of suicidal ideation, other psychological problems, and mood assessment between COVID-19 callers and non-COVID-19 callers [*n* (%)].

**Assessment**	**All callers**	**COVID-19 callers**	**Non-COVID-19 callers**	*****χ^2^*****	***p***
	**(*n* = 1,154)**	**(*n* = 541)**	**(*n* = 613)**		
Suicidal ideation				65.91	<0.001
No suicidal ideation	458 (39.7)	282 (52.1)	176 (28.7)		
Ideation without plan	552 (47.8)	207 (38.3)	345 (56.3)		
Ideation with plan	144 (12.5)	52 (9.6)	92 (15.0)		
History of suicidal behavior	239 (27.8)	100 (24.0)	139 (31.3)	5.65	0.017
Substance misuse	73 (8.9)	32 (8.0)	41 (9.7)	0.75	0.390
Severe physical illness	84 (10.2)	41 (10.3)	43 (10.2)	0.003	0.958
Chronic life events	528 (64.5)	234 (58.8)	294 (69.8)	10.89	0.001
Physical/sexual abuse	130 (16.0)	54 (13.6)	76 (18.2)	3.19	0.074
Fear of assault	148 (18.2)	52 (13.1)	96 (23.0)	13.46	<0.001
Acute life events	459 (56.2)	221 (55.7)	238 (56.7)	0.08	0.774
History of suicidal behavior of family members or friends	360 (44.4)	172 (43.5)	188 (45.2)	0.22	0.637
	**(x̄ ± s)**	**(x̄ ± s)**	**(x̄ ± s)**	***t***	***p***
Psychological distress	76.89 ± 21.42	74.33 ± 22.60	79.10 ± 20.11	−3.56	<0.001
Hopefulness	37.05 ± 30.67	41.09 ± 31.36	33.56 ± 29.65	3.84	<0.001
Severity of depression	61.58 ± 22.31	57.85 ± 23.54	65.09 ± 20.51	−4.72	<0.001
	**Median (IQR)**	**Median (IQR)**	**Median (IQR)**	***z***	***p***
Intensity of suicidal ideation^a^	40 (0,75)	0 (0,60)	50 (0,80)	−8.07	<0.001

a *Given the skewed distribution of the intensity of suicidal ideation, we used the Mann-Whitney U test*.

The changes in psychological distress, hopefulness, and intensity of suicidal ideation were defined as the scores of the three variables reported by callers at the end of the index call minus the reported scores at the beginning of the call. A comparison of the changes in the three psychological variables indicated that, after the hotline psychological intervention, both groups' psychological distress and intensity of suicidal ideation were reduced whereas hopefulness increased. There was no statistically significant difference between the two groups in terms of the changes in the psychological distress and hopefulness (see Table 4). However, the decrease of intensity of suicidal ideation in COVID-19 callers was less than that in non-COVID-19 callers (*p* < 0.001).

**Table 4:** Comparison of changes in psychological variables before and after intervention between COVID-19 callers and non-COVID-19 callers [(x̄ ± s)].

**Variables**	**All callers**	**COVID-19 callers**	**Non-COVID-19 callers**	***t/z***	***p***
	**(*n* = 1,154)**	**(*n* = 541)**	**(*n* = 613)**		
Psychological distress	−26.56 ± 24.49	−26.87 ± 24.77	−26.28 ± 24.26	0.35	0.730
Hopefulness	9.69 ± 18.58	10.45 ± 18.74	9.01 ± 18.43	1.09	0.276
Intensity of suicidal ideation^a^	0 (−50, 0)	0 (−30, 0)	−15 (−50, 0)	−5.08	<0.001

a *Given the skewed distribution of the intensity of suicidal ideation, Median (IQR) and results of the Mann-Whitney U test were reported*.

Of the 1,276 callers, 868 callers, including 417 COVID-19 callers and 451 non-COVID-19 callers, completed interviews to assess depressive symptoms. Differences between the two groups on five of the nine depressive symptoms were statistically significant, i.e., depressed mood, suicidal ideation or behavior, sleep problems, loss of energy, and worthlessness. The non-COVID-19 callers were more likely to report depressive symptoms than COVID-19 callers (see Table 5).

**Table 5:** Comparison of assessed depressive symptoms between COVID-19 callers and non-COVID-19 callers [*n* (%)].

**Features**	**All callers**	**COVID-19 callers**	**Non-COVID-19 callers**	*****χ^2^*****	***p***
	**(*n* = 868)**	**(*n* = 417)**	**(*n* = 451)**		
Depressed mood				10.23	0.006
Symptomatic	492 (56.7)	215 (51.6)	277 (61.4)		
Subthreshold	28 (32.3)	145 (34.8)	135 (29.9)		
Asymptomatic	96 (11.1)	57 (13.7)	39 (8.6)		
Diminished interest				4.96	0.084
Symptomatic	420 (48.5)	186 (44.6)	234 (52.1)		
Subthreshold	221 (25.5)	113 (27.1)	108 (24.1)		
Asymptomatic	225 (26.0)	118 (28.3)	107 (23.8)		
Suicidal ideation				37.91	<0.001
Symptomatic	631 (72.9)	264 (63.3)	367 (81.9)		
Asymptomatic	234 (27.1)	153 (36.7)	81 (18.1)		
Weight change				3.56	0.169
Symptomatic	383 (45.0)	173 (41.9)	210 (47.8)		
Subthreshold	246 (28.9)	122 (29.5)	124 (28.2)		
Asymptomatic	223 (26.2)	118 (28.6)	105 (23.9)		
Sleep problem				13.90	0.001
Symptomatic	427 (50.6)	180 (44.0)	247 (56.8)		
Subthreshold	257 (30.5)	143 (35.0)	114 (26.2)		
Asymptomatic	160 (19.0)	86 (21.0)	74 (17.0)		
Agitation or retardation				5.01	0.082
Symptomatic	289 (34.3)	125 (30.6)	164 (37.8)		
Subthreshold	199 (23.6)	100 (24.4)	99 (22.8)		
Asymptomatic	355 (42.1)	184 (45.0)	171 (39.4)		
Loss of energy				21.75	<0.001
Symptomatic	455 (54.3)	188 (46.4)	267 (61.7)		
Subthreshold	198 (23.6)	105 (25.9)	93 (21.5)		
Asymptomatic	185 (22.1)	112 (27.7)	73 (16.9)		
Worthlessness				21.62	<0.001
Symptomatic	515 (61.5)	221 (54.8)	294 (67.6)		
Subthreshold	167 (19.9)	82 (20.3)	85 (19.5)		
Asymptomatic	156 (18.6)	100 (24.8)	56 (12.9)		
Diminished thinking ability				3.81	0.149
Symptomatic	453 (54.6)	208 (52.0)	245 (57.0)		
Subthreshold	174 (21.0)	95 (23.8)	79 (18.4)		
Asymptomatic	203 (24.5)	97 (24.3)	106 (24.7)		

*Each variable contains missing values, so the sum of the callers of each symptom is less than the total number of callers*.

## Discussion

According to guidance for emergency psychological crisis intervention and the psychological support hotline issued by the National Health Commission at the early stage of the COVID-19 outbreak in China (5, 6), the hotline intervention served to disseminate public health information related to the prevention and control of COVID-19 and teach coping strategies for managing stressful events and gaining emotional relief. Although many have experienced stress due to the COVID-19 pandemic (3, 4, 9, 16, 17), it is not reasonable to assume that all callers to the psychological support hotline were distressed by the pandemic and seeking help for psychological problems as a result of COVID-19. Based on our best knowledge, this is the first study to describe the social and psychological characteristics of hotline callers with or without COVID-19-related psychological disturbance.

Results of the present study indicate that, hotline callers reporting COVID-19 related psychological disturbance are different from callers who endorse psychological problems unrelated to COVID-19. COVID-19 callers were older, highly educated, employed, and more likely to be married compared with non-COVID-19 callers. Although a higher proportion of COVID-19 callers reported depression (38.4%) than the non-COVID-19 callers, depression and psychological distress severity and the prevalence and intensity of suicidal ideation were lower among COVID-19 callers than that among non-COVID-19 callers. COVID-19 callers were less likely to be involved in interpersonal conflicts, but more likely to report work-related and financial problems, compared to non-COVID-19 callers. To some extent, different psychological concerns between the two groups of callers were associated with different social roles among different age groups. During the pandemic, difficulties of financial problems (reduced work opportunities and income) were common, and persons aged 30 year or older (often responsible for earning money and supporting a family) were more sensitive to this situation and attributed it to the COVID-19 than the younger. Although family relationship problem is one of the most involved concerns in present and previous studies (10), relative less callers linked it with the pandemic, especially among people younger than 20 years old.

Previous studies have reported that more than half of the BPSH callers report suicidal ideation and/or suicide attempts (10). During the current COVID-19 outbreak, the prevalence of suicidal ideation among non-COVID-19 callers was comparable to previous studies, whereas that of COVID-19 callers was significantly lower than non-COVID-19 callers. Furthermore, the mental health problems of COVID-19 callers were less severe than that of non-COVID-19 callers. A survey on the mental health status of mainland Chinese general population in February, 2020, has shown that all were under widespread stress, with depression and anxiety in the early stages of the COVID-19 pandemic (17). Our results suggest that the mental health problems among COVID-19 callers might reflect a psychological reaction induced by the pandemic rather than clinical mental disorders. They may inform the effective allocation of mental health support during times of public health crises.

These findings highlight the value of psychological support i.e., early public education on mental health, especially on how to cope with psychological stress induced by the pandemic in response to emergent public health crises. Specifically, hotline-based interventions should focus on delivering brief psycho-education about the common physical and mental reactions to stress, and encourage the teaching of healthy coping strategies, in the context of rapport and emotional support to reduce the stressful impact of the COVID-19. Given only 15% calls of the BPSH (608/4177, see the Figure 1) complained COVID-19 related problems, the findings also indicate that we should pay attention to non-COVID-19 callers and continue to provide high quality psychological interventions during times of public health crises.

Previous studies on hotline callers during the 2003 SARS epidemic have shown that callers' main concerns were seeking emotional support and information about the epidemic (14, 15). Consistent with these studies, in our study, the most common concern of COVID-19 callers was depression. In addition, the contagiousness of COVID-19, large number of people affected, long duration of the pandemic, and limited ability to work or go to work due to lockdown or quarantine, together contributed to a high proportion of COVID-19 callers reporting financial and work-related problems. The wide range of needs reported by callers left hotline operators ill-equipped. In addition to basic counseling skills, operators need to be trained in scientific knowledge and public health information about COVID-19, in order to effectively help callers.

There was no significant gender difference between COVID-19 and non-COVID-19 callers to the BPSH. Most callers self-identified as women during the COVID-19 pandemic, as during normal times (10, 12, 13) and after catastrophic events (15, 19, 20). That is, irrespective of major public health emergencies, women still appear more likely to call the hotline in seek for psychological counseling to help themselves, and major public health events did not increase the proportion of men making calls to psychological support hotline. Crisis intervention workers should not only *passively* wait for people to come to seek help, but should also *proactively* reach out to those in need. For example, a mass media campaign can be used to disseminate information about the disease, preventive measures, some knowledge of possible physical and psychological reactions to the pandemic, and internet-based self-help coping strategies.

COVID-19 callers were better educated and more likely to be married and employed compared to non-COVID-19 callers. This may highlight discrepancy in the utilization of free and supportive resources based on socioeconomic status (SES). Our results suggest an urgent need to further publicize and promote the hotline as an immediate and convenient psychological service for those of relatively low SES. Such services seek to promote wellness and resilience, while preventing the onset of clinical disorders and, during public health emergencies, serve as a useful source of scientific knowledge for physical health. Public health campaigns might target this group to ensure equitable access and utilization.

The findings in the present study extend our knowledge of the impacts of the COVID-19 pandemic on mental health. Previous studies reported that a large number of people were psychologically disturbed during the pandemic (1–4, 16, 17), however, results in our study indicated that the severity of psychological problem (depression, suicidal ideation etc.) due to the pandemic was slight than what we have imagined, and the COVID-19 callers reported more financial or work related problems than non-COVID-19 callers. The findings implied that, to some extent, the psychological disturbance among COVID-19 callers might be a psychological reaction to the stressors induced by the pandemic, rather than clinical mental disorders. Psychological supports, coping strategies, and public education on the COVID-19 might be important psychological intervention methods during the pandemic.

There are several limitations to the present study. First, the present study recruited hotline callers in China only, which limits the generalization of our findings to other populations. Given that our results are limited in timeframe, and other countries may have experienced a more prolonged impact of the pandemic, it is not clear whether these findings would apply in countries outside of China. Second, previous studies have reported that the COVID-19 causes increased levels of depression and anxiety in the general public (2–4, 17). Given that BPSH has historically focused on suicide prevention, our data protocols are mainly designed for depression and suicide risk and as such, neglect asking about anxiety. The present study did not collect data on anxiety, which appears especially relevant for a fear-inducing global pandemic. Third, the present study did not identify whether callers were confirmed cases, front-line healthcare workers, or other important sub-groups. This limits our exploration of the associations between characteristics and differences of the caller's personal identification and the psychological problems. Fourth, non-COVID-19 callers in this study likely experienced COVID-19 related stress, and we cannot completely disregard the potential impact of the COVID-19 on their presenting concerns. Finally, we relied on callers' self-reports, which may limit the accuracy of collected data; nevertheless, the anonymous nature of hotline may lead to increased honesty during such calls, in turn, it is difficult to describe the associations of caller's personal information and his/her primary concerns more clearly.

## Data Availability Statement

The raw data supporting the conclusions of this article will be made available by the authors, without undue reservation.

## Ethics Statement

The studies involving human participants were reviewed and approved by Ethics Review Committee of Beijing Huilongguan Hospital. Written informed consent from the participants' legal guardian/next of kin was not required to participate in this study in accordance with the national legislation and the institutional requirements.

## Author Contributions

LZ and YT designed the study and conducted data analysis, LZ, ZL, YT, MW, and NL drafted the manuscript, LZ, YW, and CW contributed to collect data. All authors contributed to the interpretation and revision of the manuscript, read and approved the final manuscript.

## Conflict of Interest

The authors declare that the research was conducted in the absence of any commercial or financial relationships that could be construed as a potential conflict of interest.
